# Perfect Fillings

**Published:** 1860-06

**Authors:** 


### PERFECT FILLINGS.

BY W. H. A.

Is it possible to produce a perfect filling?

Before answering this query, it will be well to define a little, for here as elsewhere he who is the most successful in clear definition will best succeed in instructing.

*Perfection* has two grand divisions of signification, *viz:* The *absolute* and the *relative;* the former never attained in any but its own primal and immutable state of ideal existence. And it is only perceived in the abstract—whose grandeur grows upon us as we, by degrees, begin to be able to take in more and more of its magnificence and beautiful and limitlesss proportions—precluding the possibility of its residing in relative things but in an infinitesimal degree.

As to absolute perfection of any thing, but in principle, I suppose there is no dispute among thinking practical men.—But approximate perfection, I presume, almost every one will as certainly claim the ability to attain; even those who have enveighed most severely against the term have tacitly, if not distictly, laid claim to the exercise of this last definition, and no doubt worthily.

A perfect filling would be one that restores the tooth to pristine *usefulness* and beauty—these qualities required in the degree capable of being judged of by the unaided natural powers of observation; *usefulness* comprising the necessary density to resist mechanical action and not wear away faster than the tooth-substance.

Impermeability to ponderables and imponderables in the same appreciable degree of normal structures. *Beauty* consisting in agreement of color and contour, and brilliancy as to polish with the natural parts.

These conditions fulfilled, we have substantially and practically a perfect filling. I do not say that a powerful lens might not expose a great want of perfection in color and arrangement of integral points, etc., etc. An impervious shell of indestructible material—so adapted to the walls of the cavity as exclude air and moisture from without, and the requisite stiffness to withstand the ordeals of mastication and hermetically sealed *is* a perfectly *useful* filling just so long as it retains its integrity.

I am aware that I have, in my definition, required for a perfect filling, conditions never heretofore attained in any given case—for the plain reason that we are not yet in posession of any substance capable, by present known manipulations, of attaining these ends. Some one or more of the requirements being wanting, in even our best modes, with our best means. For instance, gold is not of the desired color—the same is true of any single metal or other substance capable of resisting the fluids of the mouth. Neither is, even this otherwise admirable substance capable of transmitting the imponderables (heat and electricity) in the ratio of the natural tooth-substance; rendering it, in this respect, far from a perfect substitute, and requiring the life forces to accomodate themselves to new conditions to make success predicable in a *useful* degree.

In all substances where the *color* has been attained it has unfortunately been the only condition of a perfect filling approximated—all the other requirements being so feebly expressed as to force us to set such fillings down very far in the scale, certainly as low as fourth or lower in order of merit.

What degree of approximate perfection in a filling *is* necessary to save a tooth during the time it will probably be required for use?

This *is* a question in very deed, for so various are the circumstances of teeth in the same mouth, to say nothing of different degrees of attention as to cleanliness and care vouchsafed to them in the mouths of separate individuals, that a filling quite good enough in this respect to save a tooth in some situations, entirely fails to be of service in other positions, as to location, exposure, etc., etc.

So that we must be thrown back upon individual judgment and ability in specific cases; showing us the imperative necessity there is for us to be up to the exigencies of all possible cases that might present themselves at any period of our practice by a thorough understanding of the *principles* which always underlie *correct* practice, before offering our services to the public, if we wish to escape the unpleasant ordeal of having to make apologies or retain a suspicious silence when taken by surprises in the presentation of cases we are not prepared to encounter successfully.

I fancy I hear on all sides the plaint—"Oh, please don't say such discouraging things if you do not wish to disgust us, not only with ourselves, but also the profession, so difficult of attainment in any respectable useful degree!"

To which my reply is there is no need of any really honest, earnest mind being thus affected in view of the difficulties in the way of really useful and demonstrable advancement.—

For corroboration I need only point to the almost magic progress the profession has made within the last quarter of a century.

Who at that time had the boldness to state to his patient that "Artificial Teeth" were expected by the most sanguine to answer the purpose of "*Mastication*?" And who now would be content to call his effort a success that did not effect this desirable and necessary purpose? or what Utopian of that day dared attempt the salvation of a tooth with a cavity penetrating to any considerable degree the dentine? to say nothing of the possibility of saving it by capping, exterpation, or other means so universally successful now?

I remember very well the sage advice of the *masters* of those days. And what do you suppose it was? Simply this, "Don't risk your reputation upon any cavities except those in the grinding surfaces and they small at that."

In which advice they not only discouraged all young and inexperienced operators, but also imprisoned themselves in a narrow compass of usefulness, that has wrought untold mischief to the profession and the world and been the specious pretext to invert the order of the exercise of skill by giving undue prominence to "Artificial Dentures" by establishing a preëminent *necessity* for them, instead of boldly laying the axe at the root of the evil, and thus avoid the loss of the natural organs by *timely* operation for their *sure* salvation.

*Master* must also be *pupil*, if he be not content in the time to come to stand relatively where these old masters now stand.

For our particular calling is no exception to the great *grand rule*—of progress coming to a profession from, in the main, without its own accredited means of advancement—quite as often as in the line of anticipated acquisition.

So fellow members!—earnest workers! you need not be astonished if you wake up some fine morning and find the fogs of your "*better judgment*" cleared away and see yourselves quite capable of exercising the functions of true *master*, or demonstrative teacher of all patent principles hereunto belonging.

And although I have portrayed the profession in infant state, I am sure to hail *many*, now in that necessary condition—in a vigorous childhood! and youth! and *some*, in the plentitude of an erect manhood of Professionability! I am aware of the wrath in reserve for those who plainly tell the little boy that he is yet but a babe or child when he feels the Young America Spirit of ambition stirring within him! I also am aware that it will die out as soon as they, like him, have outgrown their small dimensions, and think more of principles than means!—of the Profession than its members! The first being absolute and enduring, the other relative and transitory.

